# Spiritual Care for Cancer Patients at the End-of-Life

**DOI:** 10.3390/healthcare12161584

**Published:** 2024-08-09

**Authors:** Gema Bacoanu, Vladimir Poroch, Maria-Gabriela Aniței, Mihaela Poroch, Eliza Maria Froicu, Bianca Hanganu, Beatrice-Gabriela Ioan

**Affiliations:** 1 2nd Internal Medicine Department, Faculty of Medicine, Grigore T. Popa University of Medicine and Pharmacy of Iasi, 700115 Iasi, Romania; gema.bacoanu@umfiasi.ro (G.B.); vladimir.poroch@umfiasi.ro (V.P.); eliza-maria.froicu@umfiasi.ro (E.M.F.); 2 Department of Palliative Care, Regional Institute of Oncology, 700483 Iasi, Romania; 3 Department of Surgery, Faculty of Medicine, Grigore T. Popa University of Medicine and Pharmacy of Iasi, 700115 Iasi, Romania; 4 Department of Preventive Medicine and Interdisciplinarity, Faculty of Medicine, Grigore T. Popa University of Medicine and Pharmacy of Iasi, 700115 Iasi, Romania; 5 Medical Oncology Department, Regional Institute of Oncology, 700483 Iasi, Romania; 6 Legal Medicine Department, Faculty of Medicine, Grigore T. Popa University of Medicine and Pharmacy of Iasi, 700115 Iasi, Romania; bianca-hanganu@umfiasi.ro (B.H.); beatrice.ioan@umfiasi.ro (B.-G.I.); 7 Institute of Legal Medicine of Iasi, 700445 Iasi, Romania

**Keywords:** spiritual care, end-of-life care, palliative care, spiritual well-being

## Abstract

Background: Spiritual care for patients at the end of life is an important element in their holistic care. The aim of this study is to assess the opinions of cancer patients with limited prognosis about the importance of faith in fighting illness and the factors contributing to a better adjustment to illness and to their self-reconciliation and spiritual well-being. Material and Methods: This study used a specially designed questionnaire for cancer patients with limited prognosis. The 30 respondents were patients with an estimated prognosis of less than 1 month, cared for in a unit with palliative and home care beds. Results: The patients emphasized the importance of family as a supporter in the fight against disease (90%), followed by faith (66.7%) and a care team (63.3%). The most common concerns expressed were related to the course of their disease, family distress, fear of death, and the Russian–Ukrainian war. Conclusion: Family and faith represent important factors in supporting and caring for a patient at the end-of-life. Patients who felt spiritually at peace and were supported in their faith by family and a priest had a better spiritual state.

## 1. Introduction

Spirituality is an important dimension for patients with advanced illness and those at the end-of-life, and assessment of this dimension is a necessary process in identifying care needs and managing symptoms as effectively as possible [1]. Palliative care is the care that provides holistic care for the patient, integrating all four dimensions of the human person (physical, psycho-emotional, social, and spiritual) into the symptom control management plan [2].

It is important for healthcare professionals to become aware of the importance of meeting the care needs of patients in all four dimensions, emphasizing the need for spiritual care for cancer patients at the end-of-life [3]. Healthcare professionals’ positive attitude toward spiritual care reduces patient suffering, increases the quality of life for patients and their families, and increases patient satisfaction with the care they receive [4].

Today’s society distinguishes between spirituality and religion. The spiritual dimension is the deepest part of the human being associated with the quality and meaning of life [5]. One of the commonly used definitions of spirituality is Renetzky’s definition: “the need to find meaning, purpose and fulfilment in life, suffering and death. The need for hope and the desire to live. The need for faith and belief in oneself, in others and a power beyond oneself or God as defined by the individual” [6].

Religion comprises a personal or institutionalized set of traditional rules, principles, values, and practices related to a particular group of people or faith, and it is the service of God or the supernatural. It is a space for the expression of spiritual distress [7]. Faith is often associated with religion and spirituality, is more personal, subjective, and deeper than organized religion, and is about relationship with God [1].

The spiritual needs of patients at the end-of-life concern the need for love (to love and be loved, to be in communion with others, to be valued and respected, to forgive and be forgiven, to share with others, and for companionship), the need for faith (to have one’s own vision of the world, the need for the transcendent, for prayer and piety, and for humility and awe), the need for hope (for meaning and purpose, and for courage, perspective, and vision), the need for virtue (of integrity, morality, ethics, character, loyalty, justice, and gratitude) and the need for beauty (in art, music, nature, balance, humor, tranquility, and meditation) [8,9].

Spiritual well-being is often described as a feeling of peace. It is a fundamental dimension of people’s overall health and well-being, and it is a good quantitative indicator of the relationship between spirituality and health. Spiritual well-being reflects the quality of relationships in four areas, namely with self, with others, with nature, and with God. All four types of relationships are interconnected, and together, they determine spiritual well-being [9,10].

Spiritual distress occurs when spiritual needs are unfulfilled, when hope and trust are lacking, when love and beauty are lacking, when there are threats to beliefs and purpose in life, and when people cannot find sources of meaning, hope, love, peace, comfort, power, and connection in life. Spiritual distress is experienced as spiritual dissonance between belief and critical events that occur in one’s life [8,9].

The social and political context can have a significant impact on patients, as well as on medical staff and the healthcare system. The Russian–Ukrainian war on Romania’s border, which was ongoing at the time of the examination data collection, can be perceived as a source of stress and worry for patients [11].

Spiritual care has positive effects on a patient’s adjustment to illness and limited prognosis. It is a source of strength and courage, and it has a transformative power on the patient, family, and healthcare professionals [12]. Spiritual well-being is correlated with higher levels of physical, emotional, and functional well-being and better quality of life, with decreased uncertainty, need for support, and reduced tensions in end-of-life care decision-making [13].

Indicators of spiritual distress lead us to observe a patient’s verbal and non-verbal behavior, where the following can be identified: feelings of hopelessness, meaninglessness, and powerlessness, feelings of guilt or shame, intense suffering, lack of God’s presence, anger at God, religion, clergy, etc., unresolved feelings about death, and fear of death [14,15].

For healthcare professionals, it is important to develop the emotional, social and spiritual skills to be part of a multidisciplinary approach in the care of patients with chronic progressive diseases. Thus, healthcare professionals will know how to recognize, assess, and respond to the spiritual needs of patients at the end-of-life [16].

The aim of this study is to assess the opinions of cancer patients with limited prognosis about the importance of faith in fighting illness and the factors contributing to a better adjustment to illness and to their self-reconciliation and spiritual well-being.

## 2. Materials and Methods

This research is a quantitative study, in which we used a specially designed questionnaire as a data collection method. To design this questionnaire, items from the literature were used, including items to highlight which factors support the struggle with disease, the people with whom patients most frequently discuss their spiritual problems, items from the Faith, Importance and Influence, Community, and Address (FICA) [17]., items from The Functional Assessment of Chronic Illness Therapy–Spiritual Well-Being (FACIT-Sp-12), and a question about study participants’ opinions on which interventions would be most appropriate to improve spiritual care. The choice of using the FICA and FACIT-Sp-12 instruments was due to their international recognition in the assessment of the spiritual dimension of patients with chronic and terminal diseases. These instruments have previously been used in similar research to assess the importance and influence of faith and spiritual well-being in chronic disease management. The associated reliability of these instruments is well documented, with Cronbach’s Alpha coefficient values above 0.7, indicating satisfactory internal consistency [18,19].

For the use of the items in the FACIT-Sp-12 instrument, we obtained the consent of the authors of the questionnaire. Refinement of the questionnaire was carried out with the help of a focus group, in which 2 physicians, 6 nurses, a priest, and a psychologist with experience in palliative care participated.

The questionnaire contains 17 items/questions collecting data on the following (Annex S1):

- Socio-demographic characteristics and disease of patients participating in the research, (gender, age, marital status, education, professional status, religion, and clinical information on disease/diagnosis)—items 1–6;
- Data on who helps them in fighting against the illness, quantitative and qualitative data on the assessment of the spiritual dimension, the importance of faith and spirituality in life and in coping with the illness, factors that give meaning to life, and the patient’s concerns or fears in fighting against the illness—items 7–14;
- Data on the assessment of the patient’s spiritual well-being (FACIT-Sp-12), and the patient’s indication of the person with whom he/she most frequently discusses his/her spiritual problems—items 15–16.

The questionnaire ends with item 17, which addresses the patient’s wishes and suggestions for actions that can improve the quality of spiritual care.

### 2.1. Selection of the Study Group

Participants were selected from patients admitted to a palliative care bed unit as well as patients cared for at home from February to May 2023. The inclusion criteria for the study were as follows: age over 18 years, patients with oncological conditions, palliative care recipients, patients who presented spiritual distress and requested spiritual interventions, patients with the ability to speak, who understood the questions, and who were willing to answer of these questions. The estimated prognosis of the study participants was estimated to be less than 1 month, for which the Palliative Prognostic Score—(PaP) was used [20]. Patients who refused to participate in the study, patients with psychiatric conditions or psychiatric manifestations, and those who did not understand the questions asked were excluded. 

#### Ethical Aspects and Methodological Barriers

The ethical challenges of this study concerned the vulnerability of the population from which the study participants were recruited; the psycho-emotional suffering of these patients, which has a major impact on the informed consent necessary for their inclusion in the study group and to ensure their protection and autonomy; and the potential conflicts of interest regarding the double role of the clinician–researcher, when medical staff is also involved in research [21]. These patients are not always able to protect their own interests as a result of a lack of decision-making capacity or because their choices may not be voluntary [22]. The defense of the patient’s interests, as well as their defense against abusive investigations and interventions, fell mainly to their palliative care provider. Another vulnerability of these patients derives from the complexity and precariousness of their physical and mental status, with general deterioration near the end-of-life. Thus, maintaining the target group and enrolling a sufficient number of people in the study was extremely difficult.

There was a possible power imbalance between the medical staff who assisted in completing the questionnaires and the patients. The staff were instructed to be as neutral as possible and to not influence the patients’ responses. The patients were encouraged to answer honestly and express their opinions freely.

The questions of the questionnaire and the purpose and finality of this research were explained to the patients, after which the patients signed the informed consent for participation in this study. To fill in the questionnaire, the participants were helped by the care staff who had been previously trained for this purpose. The participants were informed that they could withdraw from the study at any time without suffering repercussions. This was made clear both verbally and in the informed consent document.

The items of the questionnaire were explained to the patients, along with the purpose and aim of this research, after which the patients signed informed consent to participate in this study. To complete the questionnaire, the participants were assisted by nursing staff who had been previously trained for this purpose.

The study group consisted of 30 patients: 20 patients cared for in a unit with palliative care beds, and 10 patients under palliative home care.

### 2.2. Data Interpretation

The results obtained were analyzed using SPSS 26.0 software [23], performing descriptive data analysis and multiple correspondence analysis among categorical variables, such as gender, environment, current worries, and faith questions. Cronbach’s Alpha was calculated to assess the internal homogeneity of the question sets related to each aspect. Values exceeding 0.7 are considered satisfactory, implying high reliability. Values exceeding 0.8 [24,25] are considered highly satisfactory. The percentage of variance explained serves as a vital measure to determine the significance of each aspect. Aspects with a higher percentage of variance explained are frequently perceived as more relevant to the model [26]. 

### 2.3. Thresholds of Significance and Interpretation

The use of these methods and significance thresholds facilitated an in-depth assessment of how the categorical variables are associated with each other and how they vary across groups, providing valuable insights for tailored interventions and understanding the target group dynamics.

## 3. Results

Throughout February–March 2023, the questionnaire was administered to 30 palliative care patients who were selected according to the inclusion and exclusion criteria listed. The results of the study were divided into four categories: a description of the study group, aspects related to faith and spirituality, the degree of reconciliation with the disease and spiritual wellbeing, and the patients’ wishes and suggestions for increasing the quality of spiritual care (Annex S2).

### 3.1. A. Description of the Study Group (Items 1–6)

Table 1 summarizes the socio-demographic data of the study participants. The majority of participants were female (19 patients—63.3%), and 15 of the patients were over 70 years of age (50%). The age of the patients ranged from 35 to 81 years, and the mean age of the study group was 66.57 ± 11.38 years, which is close to the median value (69, 50 years), suggesting homogeneity of the series of values. The mean age was significantly lower in females than males (63.63 vs. 71.64 years; *p* = 0.028).

The majority of patients were from urban areas, with an urban/rural ratio of 2.3/1. The patients from urban areas were predominantly female (61.9%) and aged over 70 years (52.4%), while the patients from rural areas were predominantly female (66.7%) and aged under 70 years (55.6%) (Table 1).

A total of 76,7% of the subjects were Orthodox, predominantly female (56.5%; *p* = 0.179), aged over 70 years (52.6%; *p* = 0.826), and from urban areas (63,2%; *p* = 0.102). The Catholic patients were all female, with 66.7% aged under 70 years.

When asked “please, if you wish, tell us about your disease/sickness”, only 63.3% indicated that they suffered from an oncological disease. They were more frequently female (57.9%), under 70 years old (52.6%), and urban patients (73.7%).

### 3.2. B. Aspects Regarding Faith and Spirituality (Items 7–10)

Regarding “What/who helps you fight the illness?” (item 7), the most frequent response was family (90%), followed by faith (66.7%) and the care team (63.3%). Two of the patients mentioned that friends were the ones who helped them in their fight against the disease.

In response to the question “Does faith occupy an important place in your life?” (item 8), the majority (90%) considered faith to occupy an important place in their lives. Two people (6.7%) of all respondents did not consider faith to be important, and one person (3.3%) of all respondents chose not to answer the question. 

In response to the question “Is faith a support for you in your current situation?” (item 9), 26 people considered faith to be a support for them, which represents 86.7% of all responses. This is the overwhelming majority of respondents, indicating a high prevalence of faith as a form of support among them. Only two people answered that faith was not a support for them, representing 6.7% of the total responses, and two people chose not to answer this question, also constituting 6.7% of the total. This choice indicates that a small fraction of the sample either did not have a formed opinion about the role of faith as a support or chose not to express an opinion in the survey. 

Correspondence analysis between the patients’ genders and the importance of faith in the patients’ lives and faith as a support in the fight against illness suggests significant differences between men and women (see Table 2).

Dimension 1 shows excellent reliability, with an alpha of 0.826, and explains 74.174% of the variance, indicating a consistent measure of concept. Dimension 2, with an alpha of 0.635, is acceptable, explaining 57.780% of the variance, but suggests a weaker correlation between the items compared to the first dimension. The mean value of variance explained by both dimensions is 65.977%. These results highlight the varying degrees of consistency and importance of factors in the analysis.

**Figure 1:**
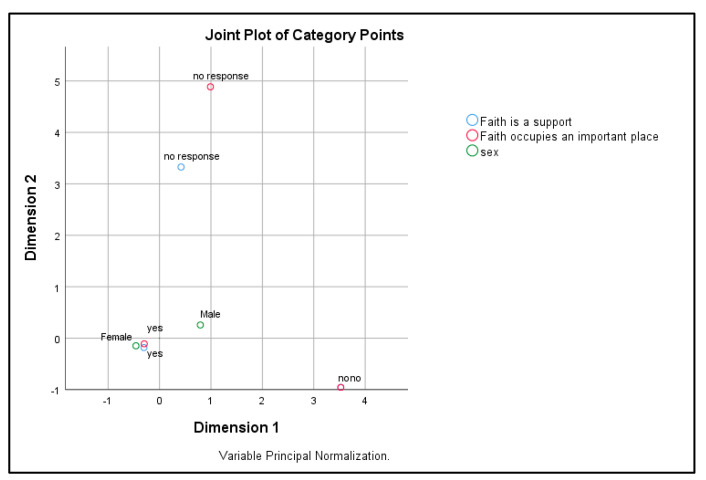
Correspondence analysis between respondents’ genders and importance of faith.

Figure 1, representing the correspondence analysis, illustrates that men tended to report faith as a support more frequently than women. Dimension 1 shows the degree to which faith was perceived as supportive, with “yes” and “no” responses at the extremes. Dimension 2 distinguishes the neutral or hesitant responses, placing them at the top. Although these are not large differences, they suggest the subtle ways in which faith is integrated differently into men’s and women’s lives (see Figure 1).

With regard to the correspondence analysis between the study participants’ backgrounds (urban/rural) and the importance of faith in life and faith as a support in struggling with illness, it is shown that there were no significant differences between the “urban” and “rural” categories (see Table 3).

Dimension 1 has an alpha of 0.771, indicating good reliability and relative consistency among questions. This explains 68.625% of the total variance, highlighting the significant influence of background on perceptions of faith. Dimension 2, with an alpha of 0.615, shows moderate internal consistency and explains 56.476% of the variance, suggesting that the factor includes a variety of aspects of faith or different interpretations by subjects. Thus, both dimensions are relevant, but the former is predominant.

**Figure 2:**
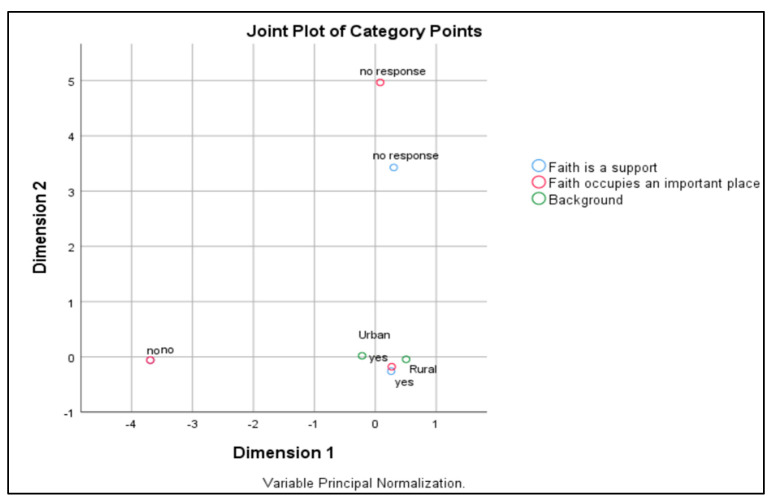
Correspondence analysis among background (urban, rural), importance of faith, and perception of faith as a support.

The chart in the multiple correspondence analysis highlights the relationships between background (urban, rural), importance of faith, and perception of faith as a support. The categories of “urban” and “rural” are almost overlapping, showing similarities among the responses. Affirmative responses about faith are concentrated, indicating a broad recognition of the importance of faith in both environments. The “no” and “I don’t answer” responses are spaced apart, suggesting different perspectives or a reluctance to discuss faith (see Figure 2).

For item I10, “Do you feel at peace with yourself?”, 19 of the subjects (63,3%) answered yes. They were more frequently women (69.2%) from urban areas (69.2%). A total of 19 people (63.3% of the total responses) indicated that they felt at peace with themselves, 8 people (26.7% of the total responses) indicated that they did not feel at peace with themselves, and 3 people (10.0% of the total responses) did not give an answer to the question.

For item I11, “What are you worried about at the moment?”, the patients were most frequently worried about their illness and its worsening (43.6%), followed by family distress (28.4%), personal distress at the end-of-life (fear of death—9%), dependence on others in the care process (31.2%), and the presence of the Russian–Ukrainian war (27.5%) (see Table 4).

**Figure 3:**
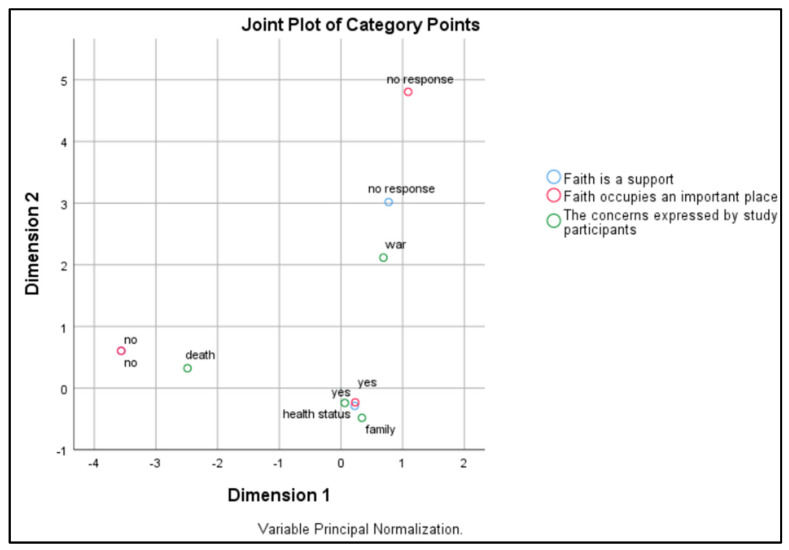
Correspondence analysis among the concerns expressed by the study participants, the powerlessness of faith in life, and faith as a support in illness.

The correspondence analysis among the concerns expressed by the study participants, the importance of faith in life, and faith as a support in the fight against illness (see Figure 3) revealed links between the patients’ concerns and their perceptions of faith. 

In contrast, those concerned about death tended to be closer to negative responses, indicating a possible lack of belief in the role of faith. Those concerned about war were in a neutral zone, indicating that, for them, the role of faith may vary or be ambiguous.

The “yes” and “no” answers were polarized, reflecting the clear differences between those who saw faith as fundamental and those who did not. The “I don’t answer” category ranks high in Dimension 2, suggesting uncertainty or a reluctance to discuss faith. Dimension 1 captures the degree of affirmation or denial of faith as supportive and important, with health and family concerns associated with a positive perception. Dimension 2 appears to indicate the levels of uncertainty or neutrality toward faith (see Figure 3). Understanding how different concerns influence perceptions of faith may facilitate more personalized and effective approaches to patient care.

### 3.3. C. Degree of Reconciliation with Illness and Spiritual Well-Being

For item I12, “What/who will help you feel at peace?”, the most frequent answers were divinity and prayer, priest and church, family and friends, and medical staff. For item I13, “What brings meaning and peace into your life?”, 63.3% of the subjects considered that family brings meaning and peace into their lives, 20% of the patients answered work and family, 3.3% answered only work, and 13.3% of the patients did not answer this question (see Table 5).

For item I14, “What could support your faith?”, 11 people considered that a priest could support their faith, 7 people considered that family could support their faith, 3 people considered that both a priest and their family could support their faith, and 9 people did not answer this question, indicating a relatively large proportion of missing values and uncertainty regarding this theme. 

Of the subjects who responded that a priest and the church could support them in their faith, 75% were female and 75% were from urban areas. Only one female patient, aged under 70, from an urban area, responded that no one could support her in strengthening her faith.

The results of the 12 statements of item I15, which is the FACIT-Sp-12 scale that assesses the spiritual well-being of the patients participating in the study, are shown in Table 6.

For every subject, the score for each statement in I15 was calculated. The theoretical score for this set of statements ranges from 0 to 48. A low score is associated with spiritual distress. The I15 score ranged from 14 to 38, recording a mean level of 29.20 ± 7.03, which is relatively close to the median value (30.50), and the results of the skewness and Kurtosis tests were in the range of [−2 ÷ +2], suggesting that the set of I15 score values is homogeneous, so statistical significance tests could be applied. 

The mean level represents 60,8% of the maximum possible. The mean score for I15 classifies the surveyed group as having moderate spiritual well-being.

The average I15 score was significantly lower in males (25.91 vs. 31.11; *p* = 0.049), and about 14% of patients showed an association of a lower I15 score with older age (r = −0.138; *p* = 0.467). The average I15 score was slightly lower in patients from urban areas (28.10 vs. 31.78; *p* = 0.194).

In fighting their disease, the patients who were helped by their care team, faith, and church, had a slightly higher I15 score. For the patients for whom faith was important and supportive, the I15 score was significantly higher. Likewise, the patients who felt at peace with their soul and were supported in their faith by their family and a priest had a significantly higher I15 score (see Table 7).

For I16, “Who do you most frequently discuss spiritual issues with?”, the top response was family and a priest, followed by a physician and the rest of the health care team (nurse, psychologist, etc.).

For I17, “What do you think needs to be changed for better spiritual care?”, only 50% of the subjects answered this question. Their responses emphasized the importance of more frequent discussions with their priest and that they would like support from the medical staff to facilitate participation in prayer or church. Others expressed regret for neglecting spiritual aspects in the past.

## 4. Discussion

The results of this study highlight the importance of faith and spiritual care in advanced cancer patients with limited life expectancy, and they are in agreement with the literature indicating that spiritual care can have an important impact on health status, particularly in terms of achieving inner peace, harmony, happiness, and coping with illness and end-of-life [3,27]. 

The spiritual well-being of the study participants was assessed, and then the interactions among various socio-demographic factors were analyzed. The study participants had a moderate degree of spiritual well-being and soul reconciliation, which derives from approaching the end-of-life, with the patients having an estimated prognosis of less than one month. Other studies have confirmed that disease progression, the deterioration of patients’ general condition, and approaching one’s end-of-life are associated with reduced spiritual well-being [28].

From the distribution of scores, it can be concluded that, in general, in both urban and rural areas, there was a clear tendency to see faith as important and supportive. However, there was also a distinct minority who rejected this idea or chose not to answer the questions, suggesting that the subject of faith may be perceived differently by different groups.

With respect to the patients’ ages and gender factor, the results revealed differences between men and women with respect to the degree of reconciliation and spiritual well-being, in the sense that men and younger patients reported a lower degree of soul reconciliation and spiritual well-being. These results are confirmed by studies showing that women and older people have a greater inclination toward spirituality and religion, tend to be more religious, and thus, have better spiritual well-being. Men and women also integrate spirituality differently [29].

The patients’ resources in the fight against their illness are family, faith, and a care team, and the most frequent people they communicate with about their spiritual problems are family members, a priest, and the physician, with family and priests as faith support resources scoring statistically significantly in our research. These results are reinforced by those in the literature that specify the need for holistic care for end-of-life patients and the need for an interdisciplinary team in palliative care [30]. Both patients and their families find psychological and spiritual support in healthcare professionals, as well as help with symptom control, communication, and decision-making at the end-of-life [31]. Close family relationships, faith in God, and religious practices, such as prayer, play a role in maintaining dignity in patients at the end-of-life [32].

The patients’ concerns expressed in this study were about their health condition, about the suffering of the family witnessing the patient’s illness, the fear of death, and the social situation of instability generated by the Russian–Ukrainian war. These illustrate that it is the illness that dominates the lives, concerns, and fears of patients at the end of their lives, but they remain anchored in the social reality in which they live. The literature also confirms the fears of advanced cancer patients, indicating that it diminishes the quality of life and induces a fear of death [33]. Other studies have also pointed out that spiritual care helps people find meaning, hope, and wholeness in their lives and relationships [9].

Patients who said that faith is an important part of their lives and a support in their struggle with illness said that they felt more at peace spiritually, showed better spiritual well-being, and if they were supported in their faith by family and a priest, they found motivation to live and accept their situation. These observations were also made by Puchalski and Romer (2000) who emphasized the role of faith and religiosity in finding meaning and purpose in life. It is through faith that people learn to cope with and understand suffering, which is an effective way of adapting to the realities and limitations of illness [34].

Given the significant impact that religion and spiritual beliefs have on the ways in which many patients cope with cancer and their approaching end-of-life, we understand the importance of healthcare professionals assessing these spiritual needs of advanced cancer patients at their end-of-life and providing the most appropriate setting for the highest-quality spiritual care.

### Limitations of this Study

- Biases: There is a possibility of biases introduced by the self-reporting of spiritual experiences and the involuntary influence of the patients’ answers by the medical staff who assisted in completing the questionnaires. These aspects may have affected the accuracy and objectivity of the results.
- Presence of Russian–Ukrainian war: The period of data collection coincided with the ongoing Russian–Ukrainian war, which was mentioned as a source of stress and anxiety by the patients. This may be a contributing factor influencing the participants’ spiritual perceptions and experiences.
- Survey limitations: The questionnaire used may not have captured all relevant aspects of spiritual well-being and spiritual care due to limitations in its design. Incomplete questionnaires were also excluded from the final analysis, which could have introduced another type of bias.
- Population: The relatively small sample size and the selection of participants from a single geographic region limit the generalizability of the results. The participants were drawn from a palliative care unit and home care patients with cancer, which may not be representative of all terminally ill patients.
- Exclusion of patients with psychiatric conditions: Patients with psychiatric conditions were excluded from the study, which may limit our understanding of spiritual well-being in this group. Exclusion was confirmed by medical evaluations carried out by medical specialists.

## 5. Conclusions

Faith and spiritual care occupy an important place in the lives of advanced cancer patients with limited prognosis. Holistic patient care in the interdisciplinary team offered by palliative care is one of the best solution is for quality end-of-life care, with integration of the spiritual domain into the care plan.

In their fight against illness, patients find support in their care team, in faith and church, in family, and their priest. Patients for whom faith is an important part of their lives and a support in their struggle with illness feel at peace, and if they have been supported in their faith by their family and priest, they find the motivation to live and accept their situation.

Knowledge of these resources for a patient fighting against illness can help caregivers and health care providers develop health care services and modify quality standards of care to provide patients with quality care adapted to all physical, psycho-emotional, social, and spiritual needs. Also, training health care professionals to understand and assess the spiritual needs of patients at their end-of-life remains a concern of any health and palliative care provider.

## Figures and Tables

**Table 1:** Socio-demographic characteristics of the study group (items 1–5).

Age (Years)		66.57 + 11.38 (35–81)	*p* = 0.028
	Urban (N = 21)	Rural (N = 9)	
<70 years	10 (47.6)	5 (55.6)	*p* = 0.690
≥70 years	11 (52.4)	4 (44.4)	
Gender (n (%) U/R)			
Male	8 (38.1)	3 (33.3)	*p* = 0.803
Female	13 (61.9)	6 (66.7)	
Level of education/studies (n (%) Urban/Rural)			
Secondary school	15 (50)	9 (30)	-
Higher education school	6 (20)	0	-
Religious affiliation (n (%))			
Orthodox 23 (76.7)			
Women 13 (56.5)	9 (69.2)	4 (30.8)	*p* = 0.179
Men 10 (43.5)	7 (70)	3 (30)	
Catholics 3 (10)			
Women 3 (100)	1 (33.3)	2 (66.7)	-
Men 0	0		
Others 4 (13.3)			
Women 3 (75)	3 (100)	0	-
Men 1 (25)	1 (100)	0	-

**Table 2:** Correspondence analysis between patients’ genders and importance of faith as support.

Model Summary				
Dimension	Cronbach’s Alpha	Variance Accounted For		
		Total (Eigenvalue)	Inertia	% of Variance
1	0.826	2.225	0.742	74.174
2	0.635	1.733	0.578	57.78
Total		3.959	1.32	
Mean	0.742	1.979	0.66	65.977

**Table 3:** Correspondence analysis between background (urban/rural) and the importance of faith in life and faith as support in illness.

Model Summary				
Dimension	Cronbach’s Alpha	Variance Accounted For		
		Total (Eigenvalue)	Inertia	% of Variance
1	0.771	2.059	0.686	68.625
2	0.615	1.694	0.565	56.476
Total		3.753	1.251	
Mean	0.701	1.877	0.626	62.550

**Table 4:** Concerns expressed by study participants.

What Is Your Current Concern					
		Frequency	Relative Frequency	Valid Relative Frequency	Cumulative Relative Frequency
Valid	State of health	19	63.3%	67.9%	67.9%
	Family distress	4	13.3%	14.3%	82.1%
	Fear of death	2	6.7%	7.1%	89.3%
	War	3	10.0%	10.7%	100.0%
	Total	28	93.3%	100.0%	
Missing values		2	6.7%		
Total		30	100.0%		

**Table 5:** Results for I13: “What brings meaning and peace into your life?”.

What Brings Meaning and Peace into Your Life					
		Frequency	Relative Frequency	Valid Relative Frequency	Cumulative Relative Frequency
Valid	Work	1	3.3	3.8	3.8
	Family	19	63.3	73.1	76.9
	Both	6	20.0	23.1	100.0
	Total	26	86.7	100.0	
Missing values		4	13.3		
Total		30	100.0		

**Table 6:** Patients’ responses to the FACIT-Sp-12 scale (I15).

Item 15	Not at All	Less	Sort of	Quite	A Lot
(a) I feel at peace/relaxed.	2 (6.7%)	2 (6.7%)	12 (40.0%)	14 (46.7%)	-
(b) I have a reason to live.	-	1 (3.3%)	7 (23.3%)	12 (40.0%)	10 (33.3%)
(c) My life has been productive.	-	3 (10.0%)	5 (16.7%)	10 (33.3%)	12 (40.0%)
(d) I’m having trouble keeping quiet.	4 (13.3%)	11 (36.7%)	11 (36.7%)	4 (13.3%)	-
(e) I feel a purpose in my life.	1 (3.3%)	5 (16.7%)	10 (33.3%)	6 (20.0%)	8 (26.7%)
(f) I’m able to reach deep inside myself for comfort.	2 (6.9%)	5 (17.2%)	10 (34.5%)	9 (31.0%)	3 (10.3%)
(g) I feel a sense of harmony within myself.	-	7 (23.3%)	9 (30.0%)	10 (33.3%)	4 (13.3%)
(j) My life has no meaning and no purpose.	11 (36.7%)	9 (30.0%)	6 (20.0%)	3 (10.0%)	1 (3.3%)
(k) I find comfort in faith.	1 (3.3%)	5 (16.7%)	4 (13.3%)	8 (26.7%)	12 (40.0%)
(l) I find strength in faith.	1 (3.3%)	4 (13.3%)	5 (16.7%)	7 (23.3%)	13 (43.3%)
(m) Illness has strengthened my faith.	1 (3.3%)	6 (20.0%)	1 (3.3%)	10 (33.3%)	12 (40.0%)
(n) No matter what happens with my illness, things will be fine.	1 (3.3%)	2 (6.7%)	10 (33.3%)	11 (36.7%)	6 (20.0%)

**Table 7:** Correlations of I15 scores with faith-related questions.

Question	Yes (+SD)	No (+SD)	Student’s *t*-Test *p*
Care team helps in the fight against illness	30.63 ± 6.87	26.73 ± 6.92	0.146
Family helps in the fight against illness	28.78 ± 6.97	33.00 ± 7.81	0.333
Faith helps in the fight against illness	30.50 ± 6.49	26.60 ± 7.74	0.156
Church helps in the fight against illness	31.58 ± 5.98	27.61 ± 7.39	0.132
Faith is important	30.26 ± 6.50	17.50 ± 2.12	0.029
Faith is a support	30.27 ± 6.63	17.50 ± 2.12	0.036
At peace	31.21 ± 5.47	24.25 ± 7.87	0.050
Family and priest support in faith	34.75 ± 4.57	23.75 ± 7.91	0.006

SD—standard deviation. Note: The levels of significance for *p* are as follows: *p* < 0.05 indicates a significant difference, and *p* < 0.01 indicates a highly significant difference.

## Data Availability

Data are contained within the article.

## Supplementary Materials

The following supporting information can be downloaded at: https://www.mdpi.com/article/10.3390/healthcare12161584/s1, The following supporting information: Annex S1 (the questionnaire used in research) and Annex S2 (table of the global evaluation of the answers to the questionnaire items) are available.
