# Supplementary material for: Spiritual Care for Cancer Patients at the End-of-Life
Source: Healthcare (Basel). 2024 Aug 9;12(16):1584. doi: 10.3390/healthcare12161584 (PMC11353481; doi:10.3390/healthcare12161584)
Supplement: Supplementary file 1 [file healthcare-12-01584-s001.zip › healthcare-3103113-supplementary.pdf]

Annex S1 Questionnaire

SPIRITUAL CARE IN PALLIATIVE CARE PATIENTS

Palliative care is the comprehensive care of people suffering from incurable diseases, easing their suffering and providing support in difficult times. Spiritual care is an integral part of this care process. In this context, we would like to hear your views on what spiritual care could be provided to better adapt to the conditions of the disease and to increase the quality of life.

Please be so kind as to answer the following questions and we assure you of the confidentiality of what you have stated:

1. AGE ....YEARS

2. GENDER : FEMALE ○ MALE ○

3. YOU LIVE IN THE ENVIRONMENT: URBAN ○ RURAL ○

4. MARITAL STATUS/EDUCATION/PROFESSION

MENTION: —----------------

5. DO YOU BELONG TO A RELIGIOUS COMMUNITY/CONFESSION?

MENTION: ....

6. PLEASE, IF YOU WISH, LET US KNOW YOUR ILLNESS/SICKNESS:

....

----

7. WHAT/WHO HELPS YOU FIGHT AGAINST THE DISEASE?

- CARE TEAM ○

-FAMILY ○

- FAITH ○

- CHURCH ○

- OTHERS: ○ MENTION: ....

8. DOES FAITH OCCUPY AN IMPORTANT PLACE IN YOUR LIFE?"

YES ○ NO ○ I DON'T ANSWER ○

9. IS FAITH A SUPPORT FOR YOU IN YOUR CURRENT SITUATION?

YES ○ NO ○ I DON'T ANSWER ○

10. DO YOU FEEL AT PEACE WITH YOURSELF?

YES ○ NO ○ I DON'T ANSWER ○

11. WHAT IS YOUR CURRENT CONCERN?

....

....

12. WHAT/WHO HELPS YOU TO FEEL AT PEACE?

----

13. PLEASE TELL ME, WHAT BRINGS MEANING AND PEACE TO YOUR LIFE?

YOUR WORK, ○

YOUR FAMILY, ○

WHAT ELSE? ○MENTION: ....

14. HOW CAN WE SUPPORT YOU IN YOUR FAITH?

PLEASE SPECIFY A FEW THINGS YOU WOULD LIKE US TO DO FOR YOU:

----

15. BELOW IS A LIST OF STATEMENTS THAT OTHER PEOPLE WITH YOUR ILLNESS HAVE MENTION AS BEING IMPORTANT. PLEASE LET US KNOW YOUR RESPONSE. BY GIVING A RATING FROM 0-4, AS YOU FEEL IT APPLIES TO THE LAST 7 DAYS OF YOUR LIFE:

0 = NOT AT ALL, 1= LITTLE, 2= SORT OF, 3= RATHER LITTLE, 4= VERY MUCH.

a). I FEEL AT PEACE/RELAXED:

0	1	2	3	4
NOT AT ALL	LITTLE	SORT OF	RATHER LITTLE	VERY MUCH
○	○	○	○	○

b). I HAVE A REASON TO LIVE

0	1	2	3	4
NOT AT ALL	LITTLE	SORT OF	RATHER LITTLE	VERY MUCH
○	○	○	○	○

c). MY LIFE IS PRODUCTIVE

0	1	2	3	4
NOT AT ALL	LITTLE	SORT OF	RATHER LITTLE	VERY MUCH
○	○	○	○	○

d). I HAVE PROBLEMS WITH MY PEACE OF MIND

0	1	2	3	4
NOT AT ALL	LITTLE	SORT OF	RATHER LITTLE	VERY MUCH
○	○	○	○	○

e). I FEEL THERE IS A PURPOSE IN MY LIFE

0	1	2	3	4
NOT AT ALL	LITTLE	SORT OF	RATHER LITTLE	VERY MUCH
○	○	○	○	○

f). I'M ABLE TO REACH DEEP INSIDE MYSELF FOR COMFORT

0	1	2	3	4
NOT AT ALL	LITTLE	SORT OF	RATHER LITTLE	VERY MUCH
○	○	○	○	○

g). I FEEL A SENSE OF HARMONY WITHIN MYSELF

0	1	2	3	4
NOT AT ALL	LITTLE	SORT OF	RATHER LITTLE	VERY MUCH
○	○	○	○	○

j). MY LIFE HAS NO MEANING AND NO PURPOSE

0	1	2	3	4
NOT AT ALL	LITTLE	SORT OF	RATHER LITTLE	VERY MUCH
○	○	○	○	○

k). FIND COMFORT IN MY FAITH OR SPIRITUAL BELIEFS

0	1	2	3	4
NOT AT ALL	LITTLE	SORT OF	RATHER LITTLE	VERY MUCH
○	○	○	○	○

l). I FIND STRENGTH IN MY FAITH OR SPIRITUAL BELIEFS

0	1	2	3	4

NOT AT ALL	LITTLE	SORT OF	RATHER LITTLE	VERY MUCH
○	○	○	○	○

m). MY ILLNESS HAS STRENGTHENED MY FAITH OR SPIRITUAL BELIEFS

0	1	2	3	4
NOT AT ALL	LITTLE	SORT OF	RATHER LITTLE	VERY MUCH
○	○	○	○	○

n). I KNOW THAT WHATEVER HAPPENS WITH MY ILLNESS, THINGS WILL BE FINE.

0	1	2	3	4
NOT AT ALL	LITTLE	SORT OF	RATHER LITTLE	VERY MUCH
○	○	○	○	○

16. With whom do you most frequently discuss spiritual issues?

- family ○

- physician ○

- nurse ○

- psychologist ○

- priest /spiritual assistance/ ○

- Other. ○ Mention: ....

17. What do you think should be changed or improved for better spiritual care?

THANK YOU AND WE ASSURE YOU OF OUR RESPECT AND APPRECIATION!

Annex S 2. Table of global evaluation of the answers to the questionnaire items

Variable	Category	Frequency	Relative frequency
Gender	Male	11	36.70
	Female	19	63.30
	Total	30	100
Environment of provenience	Urban	21	70
	Rural	9	30
	Total	30	100
What is your current concern	Health state	19	63.3
	family	4	13.3
	death	2	6.7
	war	3	10
	Total	28	93.3
	Missing values	2	6.7
	Total	30	100
Is faith important?	yes	27	90
	no	2	6.7
	I do not answer	1	3.3
	Total	30	100
Faith is a support	yes	26	86.7
	no	2	6.7
	I do not answer	2	6.7
	Total	30	100
I find comfort in my faith	Not at all	1	3.3
	Little	5	16.7
	Sort of	4	13.3
	Rather less	8	26.7
	Very much	12	40
	Total	30	100
I find strength in my faith	Not at all	1	3.3
	Less	4	13.3
	Sort of	5	16.7
	Rather little	7	23.3
	Very much	13	43.3
	Total	30	100
My illness has strengthened my faith	Not at all	1	3.3

	Less	6	20
	Sort of	1	3.3
	Rather little	10	33.3
	Very much	12	40
	Total	30	100
